# Two-stage mixotrophic cultivation for enhancing the biomass and lipid productivity of *Chlorella vulgaris*

**DOI:** 10.1186/s13568-017-0488-9

**Published:** 2017-10-10

**Authors:** Hongwu Cui, Fanping Meng, Feng Li, Yuejie Wang, Weiyan Duan, Yichen Lin

**Affiliations:** 1 Key Laboratory of Marine Environment and Ecology, Ministry of Education, Ocean University of China, Qingdao, 266100 Shandong Province China; 2 National Ocean Technology Center, Tianjin, 300112 China; 3 Department of Environmental Science and Engineering, Ocean University of China, No. 238, Songling Road, Qingdao, 266100 China

**Keywords:** *Chlorella vulgaris*, Biomass, Lipid productivity, Two-stage cultivation, Fatty acid component

## Abstract

This study proposes a two-stage mixotrophic process for cultivating *Chlorella vulgaris*. Heterotrophic growth is the dominant step in Phase I (to increase microalgal biomass) and photoautotrophic growth occurs in Phase II (to improve biomass concentration and lipid production). The results show that the addition of the low-cost antioxidant sodium erythorbate (8 g L^−1^) significantly accelerates the growth of microalgae in the first stage with air aeration. Furthermore, a higher CO_2_ fixation rate was obtained in the second stage (at least 344.32 mg CO_2_ L^−1^ day^−1^) with 10% CO_2_ aeration. This approximately corresponds to an increase of 177% over simple photoautotrophic cultivation with 10% CO_2_ aeration during the whole period. The two-stage cultivation strategy achieved a maximum *C. vulgaris* biomass concentration of 3.45 g L^−1^ and lipid productivity of 43.70 mg L^−1^ day^−1^, which are 1.85 and 1.64 times those arising due to simple photoautotrophy, respectively. Moreover, an analysis of the product’s fatty acid profile indicates that *C. vulgaris* might be an ideal candidate for two-stage mixotrophic cultivation of a renewable biomass for use in biodiesel applications. Another interesting point to note from the study is that it is an insufficiency of N and CO_2_ that probably limits the further growth of *C. vulgaris*.

## Introduction

The rapid development of human society and the inexpedient consumption of fossil fuels have caused an energy crisis. At the same time, huge amounts of greenhouse gases have been emitted into the atmosphere, which have induced unexpected climate changes and global warming. Among these greenhouse gases, carbon dioxide (CO_2_) is the primary contributor to global warming, accounting for 52% (Wilbanks and Fernandez 2014). On June 5, 2017, the global monthly mean CO_2_ concentration reached 406.05 ppm, which is much higher than the 280 ppm level encountered before the Industrial Revolution (ESRL 2017). The development of cost-effective technologies for achieving CO_2_ fixation is therefore one of the major challenges that we are faced with today (Kaiwan-arporn et al. 2012).

Technologies for the sequestration and storage of CO_2_, e.g. mineral carbonation and biological fixation by plants and algae have already been developed (Toledo-Cervantes et al. 2013). Microalgae are the fastest growing plants on earth (able to grow 10–50 times faster than terrestrial plants) and also have a very high CO_2_ fixation rate (Chen et al. 2011). At the same time, microalgae constitute a source of many high-value products, e.g. biofuels, food additives, polyunsaturated fatty acids, natural colorants, health-care products, and so on. So it is generally accepted that the combination of CO_2_ bio-fixation and biofuel production using microalgae is a promising way of realizing a sustainable method of CO_2_ mitigation (Toledo-Cervantes et al. 2013). Indeed, the biodiesels that are obtained via transesterification of the lipids from microalgae have recently received widespread attention because of their non-toxicity, renewability, and environmental friendliness (Yilancioglu et al. 2014). Oleaginous microalgae potentially have great advantages as promising resources for biodiesel production: high photosynthetic efficiency, high lipid productivity per unit of land, high growth rate, and they can also be grown in a wide range of environments (Rawat et al. 2013; Wang et al. 2008). Nevertheless, the scalability, sustainability, and cost-efficiency of using large-scale algae cultivation for biodiesel production remain unproven and somewhat controversial (Toledo-Cervantes et al. 2013). The major issues to resolve are finding a cultivation system that has high cell density with optimum lipid content that can also be carried out on a suitably large scale (Huerlimann et al. 2010).

Currently, photoautotrophic cultivation is the most commonly-used strategy for cultivating microalgae (as most microalgae are autotrophs). However, some microalgae can grow with organic carbon substances simultaneously. It is well known that cultivating microalgae in heterotrophic or mixotrophic conditions is an effective way of increasing cell density, as well as lipid content, when organic carbons, e.g. sugars and organic acids, are used as carbon sources (Najafabadi et al. 2015). Studies have shown that some microalgae have higher growth rates and biomass yields (and even lipid content) under mixotrophic compared to photoautotrophic conditions. Bhatnagar et al. (2010) have investigated the mixotrophic cultivation of *C. minutissima*. They concluded that mixotrophic growth results in the production of 4.43 times more biomass than photoautotrophy if 10 g L^−1^ glucose is added. Similarly, the lipid content under mixotrophic conditions was 1.18 times that under photoautotrophic conditions. Liang et al. (2009) studied the growth rate and lipid content of *C. vulgaris* (UTEX 259) cultivated under mixotrophic conditions and showed that the biomass concentration due to mixotrophic growth reached 1.70 g L^−1^. This should be compared to 0.25 g L^−1^ produced by photoautotrophic growth with the same concentration of glucose added. Conversely, the lipid content of *C. vulgaris* in the phototrophy treatment reached up to 38% compared to 21% in the mixotrophy treatment. Furthermore, research has revealed that the antioxidant sodium erythorbate (NaE) has a better promotional effect on the growth of *Chlorella vulgaris* than glucose (Cui et al. 2017).

Therefore, in this study, a new two-stage mixotrophic cultivation process is proposed to cultivate *C. vulgaris* in order to enhance biomass concentration and lipid production. The method involves the addition of NaE in the first stage to promote a rapid growth in biomass. In the second stage, higher CO_2_ fixation rates and lipid productivities are obtained using 10% CO_2_ aeration. Finally, biomass productivity, CO_2_ bio-fixation rate, and the fatty acid (FA) profile of the microalgal lipids were analyzed to evaluate the effectiveness of the new strategy and assess its potential application to biodiesel production.

## Materials and methods

### The microalgal strain and culture medium

The microalgae strain *Chlorella vulgaris* FACHB-960 was employed in this study. It was acquired from the Institute of Hydrobiology (FACHB-collection), which is part of the Chinese Academy of Sciences in Wuhan, China. The microalgae were pre-cultured in 500-mL Erlenmeyer flasks using Bristol’s solution (Cheng et al. 2013). Continuous illumination (60 μmol photons m^−2^ s^−1^) was applied for 5 days using cool-white fluorescent tubes (F25T8/TL 950, Philips Ltd., The Netherlands) at 25 °C. The Erlenmeyer flasks were shaken six times daily by hand to avoid sticking. Algal cells in the exponential growth phase were used for the following experiments.

### Photobioreactors and chemicals

**Fig. 1:**
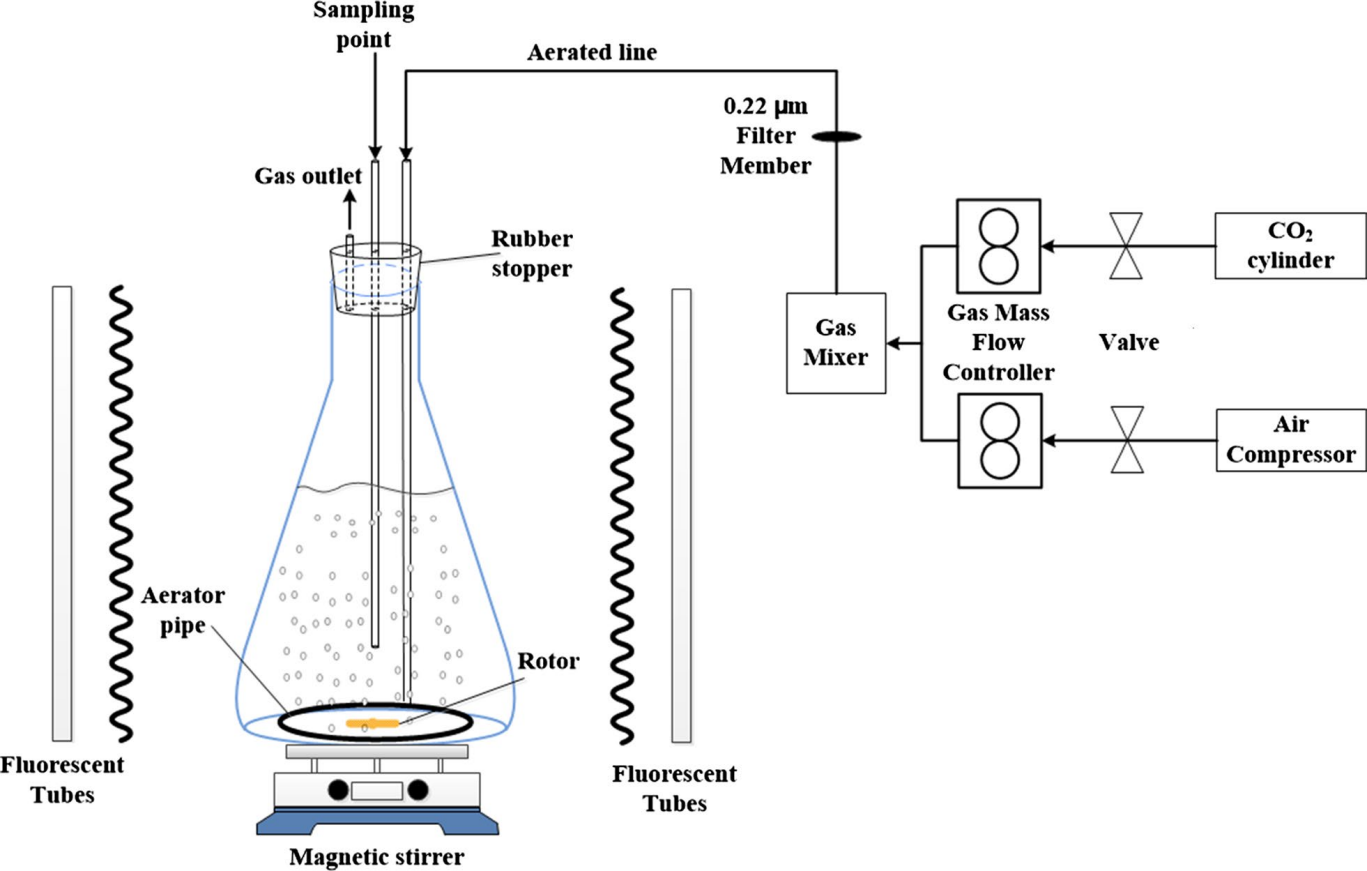
Schematic diagram of the experimental set-up and instrumentation used

The laboratory-scale airlift photobioreactor (PBR) used for *C. vulgaris* cultivation is shown in Fig. 1. The PBR has a working volume of 3 L and features a sampling point and gas inlet/outlet tubes (the former connected to the aeration apparatus). Magnetic stirrers (JB-2A, Shanghai Rex Instrument Factory, China) were positioned under the flasks to stop the algal cells sticking.

The NaE (C_6_H_7_NaO_6_·H_2_O, purity ≥  98.0%) used was obtained from the Tokyo Chemical Industry Co., Ltd. (Shanghai, China) as a yellowish powder with a solubility in water of 17 g per 100 mL.

### Experimental procedure

*Chlorella vulgaris* cells (in the exponential phase of growth) were separately inoculated into 12 PBRs with 3 L Bristol’s solution, yielding an initial cell density of 5 × 10^6^ cells mL^−1^ (an initial optical density OD_680_ of 0.25). The PBRs were divided into 4 groups: a mixotrophic group (‘MX’), mixotrophic group with nitrogen deficiency (‘MX-N’), mixotrophic group with CO_2_ deficiency (‘MX-C’), and photoautotrophic group (‘PA’). Each group consists of 3 replications. The PBRs were illuminated for 24 h (120 μmol photons m^−2^ s^−1^) using fluorescent tubes in light incubators (GXZ-280B, Ningbo Jiangnan Instrument Factory, China) at 25 °C. Details of the experimental conditions employed are given in Table 1.

**Table 1:** The conditions used in the two-stage *C. vulgaris* cultivation experiments

Group	Phase I (0–9 days)			Phase II (10–18 days)		
	NaE concentration (g L^−1^)	Aeration^a^ medium	Nutrient^b^ added on day 4 (mL)	Added carbon source	Aeration^a^ medium	Nutrient^b^ added on day 9 (mL)
MX	8	Air	6	–	10% CO_2_ (v/v)	30
MX-N	8	Air	6	–	10% CO_2_ (v/v)	0
MX-C	8	Air	6	–	Air	30
PA	0	10% CO_2_ (v/v)	6	–	10% CO_2_ (v/v)	0

–, no action

^a^Using a flow rate of 60 mL min^−1^

^b^NaNO_3_ of concentration 250 g L^−1^

#### Phase I—mixotrophic cultivation to improve biomass

Phase I was carried out to rapidly improve the biomass of *C. vulgaris* via mixotrophic cultivation. A previous study has shown that maximum *C. vulgaris* biomass is obtained if an NaE dosage of 8.0 g L^−1^ is used (Cui et al. 2017). Hence, 24.0 g solid NaE was added into 3 L Bristol’s solution of nine PBRs at the beginning of the cultivation process (i.e. groups MX, MX-N, and MX-C), achieving 8.0 g L^−1^ of NaE for each PBR. These were aerated for 9 days (using 60 mL min^−1^ air). The control group PA was continuously aerated with 10% CO_2_ (v/v) during the whole of the Phase I cultivation stage (using the same flow rate used in the other flasks). According to some pre-test results (data not shown), nitrogen deficiency is the primary nutrient limitation of Bristol’s medium. As a result, 6 mL of 250 g L^−1^ NaNO_3_ (a component of Bristol’s medium) was injected into each of the 12 PBRs on day 4.

#### Phase II—mixotrophic cultivation to improve CO_2_ bio-fixation and lipid productivity

The purpose of Phase II is to investigate the effect on biomass, lipid productivity, and fatty acid composition of the *C. vulgaris* of changing the aeration medium from air to 10% CO_2_ (assuming there is sufficient nitrogen available). At the beginning of Phase II (day 10), 30 mL of 250 g L^−1^ NaNO_3_ was added to groups MX and MX-C, and the aeration medium was changed from air to 10% CO_2_ at the same time for groups MX and MX-N until the end of the cultivation procedure.

### Analytical methods

#### Optical density and pH

The algal solutions were sampled via the sampling points in order for certain indices to be measured. The value of the optical density (OD) of each culture was measured every day using a UV–Vis spectrophotometer (model TU-1810, Persee, China). The absorbance at 680 nm was determined after the sample was appropriately diluted with deionized water to make the absorbance less than 1.0. Another 5 mL algal solution was sampled and centrifuged each day to allow the solution’s pH value to be determined (at the same height in each suspension) using a pH electrode connected to a pH meter (PHS-3C, Shanghai LIDA Instrument Factory, China).

#### Biomass, biomass productivity, carbon content, and CO_2_ fixation rate

The biomass concentration was estimated by filtering a sample through a 0.45 μm cellulose acetate membrane and drying it at 105 °C to constant mass. Biomass determination was carried at the end of Phases I and II.

The biomass productivity, *P* (mg L^−1^ day^−1^), is defined as:

1 $$ P = \frac{{X_{t} - X_{0} }}{t} $$

where *X*_0_ is the initial biomass concentration (mg L^−1^) and *X*_*t*_ is the biomass concentration at time *t* (day).

The carbon content of the biomass was determined using a CHNS analyzer (Series II 2400 CHNS/O Perkin Elmer, Boston, USA). The CO_2_ fixation rate, $P_{{{\text{CO}}_{2} }}$, can subsequently be calculated using the expression (Toledo-Cervantes et al. 2013):

2 $$ P_{{{\text{CO}}_{2} }} = C_{\text{C}}  P \left( {\frac{{M_{{{\text{CO}}_{2} }} }}{{M_{\text{C}} }}} \right) $$

where *M*_C_ is the atomic weight of carbon, $M_{{{\text{CO}}_{2} }}$ is the molecular weight of CO_2_, and *C*_C_ is the carbon content of the biomass (g_C_/g_biomass_).

#### NO_3_-N, total phosphorus, and total organic carbon concentrations

To determine the water quality, a sample of microalgae culture was centrifuged (at 4000*g* for 10 min) and the supernatant filtered through a 0.45 μm membrane. This was used to determine the NO_3_-N and total phosphorus (TP) content on days 0, 6, 16, and 18, according to the standard testing methods suggested by the Chinese state (Xu et al. 2015).

The total organic carbon (TOC) concentration was also determined using a total organic carbon analyzer (TOC-Vcpn, Shimadzu, Japan) after a culture sample was centrifuged (at 4000*g* for 10 min) and filtered through a 0.45 μm glass-fiber filter membrane (on days 0, 9, 16, and 18). Potassium hydrogen phthalate was used as a standard to calculate the TOC concentration (mg L^−1^).

#### Lipid content, lipid productivity, and fatty acid component analysis

The total lipid content was extracted using an organic solvent and determined gravimetrically. The microalgal solution was centrifuged (at 4000*g* for 10 min) at 4 °C. The resulting microalgal pellet was washed twice with distilled water, centrifuged to remove salts, and freeze dried at – 50 °C. Lipids were extracted from the dry biomass pellets using a modified solvent-based method derived from the work of Bingh and Dyer (1959). In brief, 100 mg of dried algal powder was transferred to 7.6 mL of a mixture of chloroform/methanol/water (2.5/5/2 v/v) and homogenized (Ultrasonic Homogenizer JY92-II, SCIENTZ, China) for 120 s (700 W, 5 s working, 5 s resting). The mixture was then left at room temperature for 24 h. After adding 1 mL of chloroform and 1 mL of water, the mixture was centrifuged (at 4000*g* for 10 min) to form three layers. The lowermost (organic) layer was carefully collected in a pre-weighed 10 mL glass tube and the organic solvent removed using a nitrogen evaporator. The entire extraction process was repeated twice. The total percentage lipid content, *η*, was subsequently calculated according to:

3 $$ \eta = \frac{{W_{1} }}{{W_{0} }} \times 100\% , $$

where *W*_1_ and *W*_0_ are the lipid mass (g) and dry cell mass (g), respectively.The lipid productivity, *LP* (mg L^−1^ day^−1^), of the microalgae could then be deduced using the expression:

4 $$ LP = P  \eta . $$

The FA composition of the lipids was determined via gas chromatography–mass spectrometry (GC–MS) analysis (Kebelmann et al. 2013). Freeze-dried algae samples (20–50 mg) were weighed into clean glass centrifugal tubes (10 mL) and 1 mL of saturated KOH/CH_3_OH solution added. Transesterification was carried out in a water bath (at 80 °C for 1 h). After cooling, 3 mL of distilled water and 2 mL of hexane were added and the mixture vortexed for 2 min. The upper phase containing the FAs was carefully collected and analyzed via GC–MS (7890A/5975C, Agilent, USA) using a DB-5 ms chromatographic column (50 m × 250 μm × 0.25 μm). The oven temperature was set to 80 °C, then raised to 300 °C at a rate 10 °C/min, and held at 300 °C for 5 min; the injector temperature was set to 300 °C. The helium carrier gas was supplied at a flow rate of 1 mL min^−1^. All the analyses were carried out in triplicate and the reported values correspond to their averages.

#### Statistical analysis

Statistical analysis was performed using the SPSS 17.0 software package (SPSS Co., USA). For each treatment, the means and standard deviations were calculated using the three biological replicates.

## Results

### Microalgal growth and pH variation

**Fig. 2:**
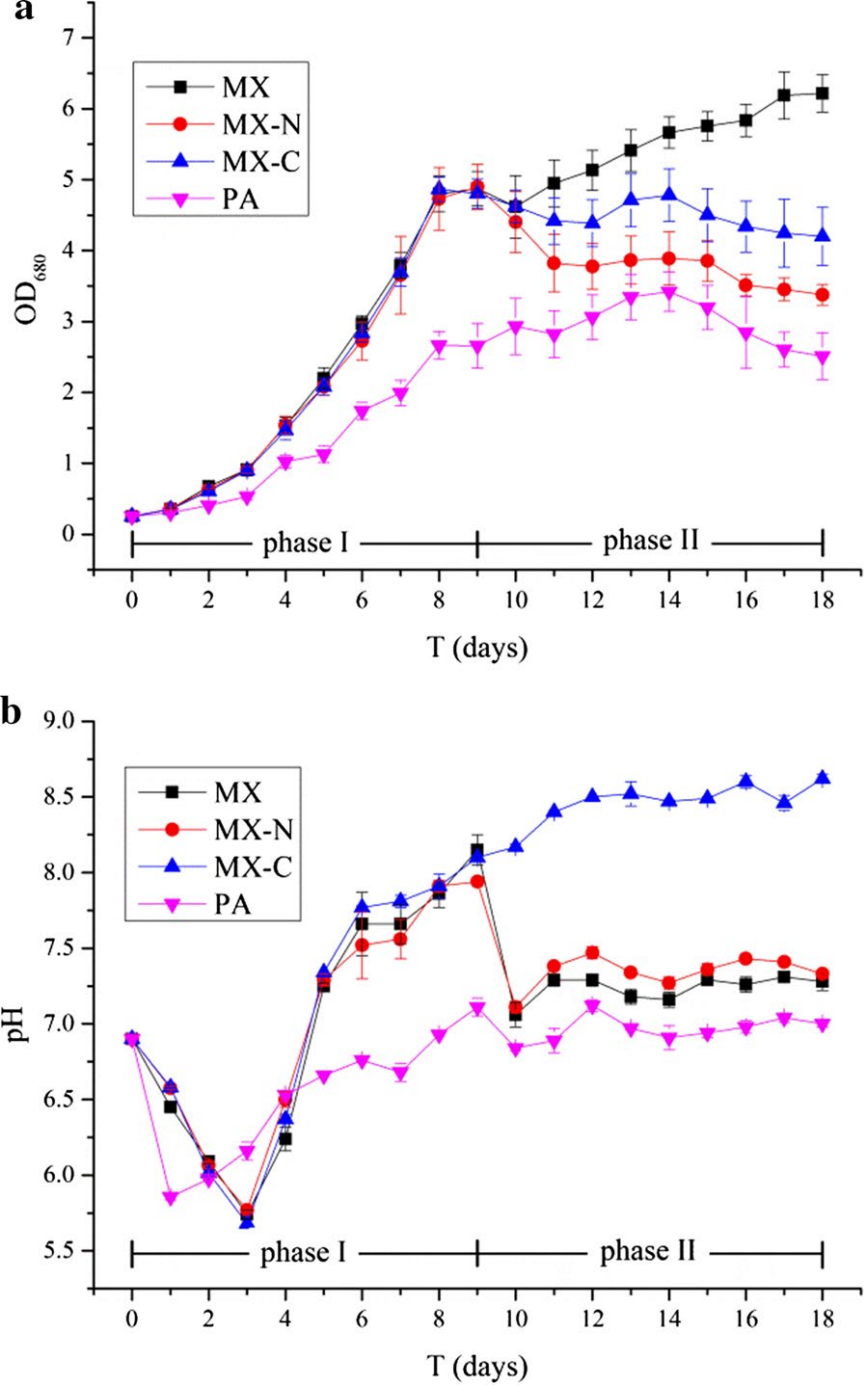
Plots showing the time-dependent changes observed in: **a** the growth of *C. vulgaris*, and **b** pH of the culture during the cultivation period for the four treatment groups. Experiments were conducted in triplicate, and the results shown are the means (with standard deviations, *n* = 3)

Figure 2a presents the growth curves obtained for the 4 groups during the cultivation period. The addition of NaE can be clearly seen to have significantly accelerated the growth of *C. vulgaris* in Phase I as the amount of *C. vulgaris* in MX, MX-N, and MX-C groups increased at a much faster rate from day 1 (with no signs of a phase lag) compared to group PA. A stationary phase was observed on day 8 in the three mixotrophic groups in Phase I in which the OD_680_ value remained around 4.8 (~ 1.8 times that observed in the control PA).

The change in conditions on day 9 lead to the growth curves of the three mixotrophic groups in Phase II exhibiting obvious differences (although their OD values remained above those of the photoautotrophic group in each case). Different levels of decline in growth of the MX, MX-N, and MX-C groups were observed on day 10. After a slight drop, and the changes in the MX-N and MX-C groups became much less obvious throughout the rest of the cultivation period. In contrast, sustained growth continued to be observed in the MX group from day 11. At the end of the cultivation period, the OD_680_ values of the 4 groups were ranked, in descending order, as: MX (6.22) > MX-C (4.20) > MX-N (3.38) > PA (2.51).

Plots of the changes in the pH values of the culture mixture are shown in Fig. 2b. A rapid decline in pH can be seen to occur at the beginning of Phase I in each group. The decline lasted for about three days in the three mixotrophic groups and just one day in the PA group (falling to about 5.73 and 5.86, respectively). After falling, the pH values in the MX, MX-N, and MX-C groups underwent dramatic increases which continued up to the end of Phase I. In one group, MX-C, the rise in pH continued right up to the end of the cultivation period (the final value was 8.62 on day 18). In contrast, sharp drops (~ 0.98) were observed in the pH values of MX and MX-N on day 10. After this drop, their pH values underwent slight increases to the end of the cultivation period, reaching 7.28 (MX) and 7.33 (MX-N) on day 18. However, the pH value in the PA group of samples ‘rebounded’ from the second day onwards, reaching 7.11 at the end of Phase I, and then remaining mostly unchanged (with some fluctuation) until the end of the experiment.

### Nutrient and TOC variation

The results obtained for the residual NO_3_-N, TP, and TOC concentrations during the two phases are shown in Table 2. We recall that 6 mL of 250 g L^−1^ NaNO_3_ was added to each PBR on day 4—this corresponds to a concentration increase of ~ 82.35 mg L^−1^ NO_3_-N for each vessel (indicated in the table using the notation ‘+ 82.35’ on day 4, assuming there was 3 L of liquid in each PBR).

**Table 2:** The variation of the nutrient and TOC concentrations in the mixotrophic and photoautotrophic culture systems

*T* (d)	NO_3_-N concentration (mg L^−1^)				TP concentration (mg L^−1^)				TOC concentration (mg L^−1^)			
	MX	MX-N	MX-C	PA	MX	MX-N	MX-C	PA	MX	MX-N	MX-C	PA
0	47.49 ± 1.07				53.92 ± 1.26				2630.00 ± 13.21		6.00 ± 0.89	
4	+ 82.35				–				–			
6	32.21 ± 1.63	37.33 ± 0.72	37.53 ± 0.49	78.62 ± 0.94	22.61 ± 0.32	24.52 ± 0.36	24.07 ± 0.43	31.96 ± 0.32	–			
9	+ 411.78	+ 0	+ 411.78	+ 0	–				1002.26 ± 30.50	990.31 ± 74.06	1007.97 ± 13.35	33.80 ± 0.75
16	333.31 ± 3.39	9.78 ± 0.84	387.66 ± 3.97	74.66 ± 0.34	7.04 ± 0.15	16.35 ± 0.15	12.48 ± 1.03	21.23 ± 0.00	968.25 ± 39.40	939.23 ± 31.69	825.40 ± 68.99	124.25 ± 4.67
18	328.86 ± 2.97	3.64 ± 0.58	378.46 ± 3.21	39.05 ± 2.71	4.71 ± 0.43	16.04 ± 0.59	11.91 ± 0.15	16.95 ± 0.73	945.02 ± 16.67	949.05 ± 28.95	602.43 ± 53.98	117.55 ± 4.21

Table 2 shows that there was a much higher utilization of NO_3_-N and TP in the three mixotrophic groups during the first 6 days of Phase I compared to the PA group. On average, the three mixotrophic groups consumed about 94.15 mg L^−1^ of NO_3_-N which is much higher than the initial NO_3_-N concentration (47.49 mg L^−1^). However, NO_3_-N and TP consumption in the PA group was much less (so the concentrations of these components were about 2.20 and 1.35 times those in the mixotrophic groups, respectively, on average).

As a large amount of NO_3_-N was consumed in the MX, MX-N, and MX-C flasks, the nitrogen content in some of these flasks (MX and MX-C) were further enhanced at the beginning of Phase II (by adding 30 mL of 250 g L^−1^ NaNO_3_, corresponding to a concentration increase of about 411.8 mg L^−1^ NO_3_-N in each flask). None was added to the MX-N flasks as these are intended to serve as ‘nitrogen-starved’ controls. The NO_3_-N concentrations at the end of Phase II were about 328.86, 3.64, 378.46, and 39.05 mg L^−1^ in groups MX, MX-N, MX-C, and PA respectively (Table 2). Clearly, there were high concentrations of NO_3_-N left in the flasks in groups MX and MX-C at the end of Phase II, but very little was left in the nitrogen-starved MX-N flasks. However, the TP concentrations in the MX and MX-C flasks (i.e. with added NaNO_3_) were relatively low at the end of the cultivation period (see Table 2), especially in the MX group where the TP concentration was found to correspond to the lowest value measured: 4.71 mg L^−1^.

Due to the addition of the NaE, the initial TOC concentration in the mixotrophic groups was 2630.0 mg L^−1^. However, their TOC values declined significantly by the end of Phase I, decreasing to ~ 1000 mg L^−1^ in each case (a utilization rate of ~ 62%). In contrast, the TOC utilization rate varied markedly in Phase II due to the changes made in the culturing conditions used. The TOC concentrations at the end of the experiment corresponded to 945.02, 949.05, and 602.43 mg L^−1^ in groups MX, MX-N, and MX-C, respectively (utilization rates of 2.2, 1.6, and 15.4%, respectively). Interestingly, the almost negligible TOC concentration in the PA group measured at the beginning of Phase I (6.00 mg L^−1^) was increased to 117.55 mg L^−1^ by the end of the cultivation period.

### Biomass concentration and productivity

The biomass results are shown in Table 3. Clearly, the biomass concentrations in the three mixotrophic groups were much higher (by roughly 164%) than that in the photoautotrophic group at the end of Phase I. Furthermore, the average biomass productivity in the mixotrophic groups was 0.18 g L^−1^ day^−1^ compared to 0.11 g L^−1^ day^−1^ in the PA group.

**Table 3:** Comparison of the change in biomass concentration, biomass productivity, lipid content, lipid production, and lipid productivity of *C. vulgaris* in the four culture systems

	MX	MX-N	MX-C	PA
Change in biomass concentration in Phase I (g L^−1^)	1.66 ± 0.05	1.59 ± 0.08	1.62 ± 0.06	0.99 ± 0.04
Change in biomass concentration in Phase II (g L^−1^)	1.79 ± 0.04	–0.13 ± 0.04	0.40 ± 0.06	0.22 ± 0.04
Biomass productivity in Phase I (g L^−1^ day^−1^)	0.18 ± 0.01	0.18 ± 0.01	0.18 ± 0.01	0.11 ± 0.00
Biomass productivity in Phase II (g L^−1^ day^−1^)	0.20 ± 0.00	–	0.044 ± 0.01	0.024 ± 0.00
Final lipid content, *η* (%)	22.8 ± 0.8	23.3 ± 1.1	20.2 ± 1.2	24.6 ± 0.9
Final lipid production (mg L^−1^)	786.60 ± 27.21	340.47 ± 21.40	407.83 ± 21.55	298.02 ± 25.61
Final lipid productivity (mg L^−1^ day^−1^)	43.70 ± 1.51	18.92 ± 1.19	22.66 ± 1.20	16.56 ± 1.42

‘–’ is used to imply the numerical calculation is meaningless here

In Phase II, the biomass variation was quite different in each group. All of the groups increased except for MX-N (see Table 3). In this nitrogen-starved group, the biomass concentration declined by 0.13 g L^−1^ during Phase II (although a slight increase was observed from day 9 to 14—see Fig. 2a). The maximum final biomass concentration was obtained in group MX and corresponded to 3.45 g L^−1^ (see Table 3). This figure is 136, 70.8, and 185% greater than the biomasses in the MX-N, MX-C, and PA groups, respectively. A rise in biomass productivity in Phase II was also found only in the MX case. In contrast, the biomass productivity in the MX-C and PA groups declined significantly in Phase II (Table 3).

### Lipid content, lipid productivity, and fatty acid composition

The two-stage procedure was carried out in order to increase lipid productivity. The results obtained for lipid production are also shown in Table 3. The lipid contents of the four groups, at the end of Phase II, were found to be of the order of 20–25% of the dry cell weight. The highest lipid content (24.6%) occurred in the PA group but, unfortunately, this group also has the lowest biomass productivity. Hence, PA has the smallest final lipid productivity value (16.56 mg L^−1^ day^−1^). The lipid content of the MX group (22.8%) is close to the average level of the four groups, but its lipid productivity is the largest observed (131.0, 92.9, and 163.9% larger than that produced in the MX-N, MX-C, and PA groups, respectively) due to the greater amount of biomass produced.

The FA compositions at the end of Phase II are presented in Table 4. As can be observed, the primary FA components are C16:0 (palmitic acid, 28.9%), C16:1 (palmitoleic acid, 24.82%), C18:1 (oleic acid, 20.59%) and C16:0 (palmitic acid, 17.26%) for groups MX, MX-N, MX-C, and PA, respectively. The percentage total saturated fatty acid (TSFA) and monounsaturated fatty acid (MUFA) both have their maximum values in the PA group. However, no polyunsaturated fatty acids (PUFAs) were found in the PA group. In contrast, PUFAs constitute large proportions of the three mixotrophic groups (i.e. those with added NaE). With respect to omega 3 (linolenic acid) and omega 6 (linoleic) FAs (also known as ‘essential’ FAs), these were found to be in high concentration in the three mixotrophic treatments. For omega 3, the percentage content corresponds to 19.79, 12.23, and 10.47%, in MX, MX-N, and MX-C, respectively. The corresponding figures for omega 6 are 2.07, 12.38, and 10.45%. C16 and C18 are the two most predominant carbon chain lengths found to occur in the FAs determined, accounting for 91.47, 87, and 79.9% of the acids in MX, MX-N, and MX-C, respectively.

**Table 4:** Relative abundances of various fatty acids at the end of the cultivation period (%)

Fatty acid	MX	MX-N	MX-C	PA
C16:0	*28.9* *±* *0.3*	15.1 ± 0.2	8.7 ± 0.2	*17.3* *±* *0.3*
C16:1	25.2 ± 0.2	*24.8* *±* *0.4*	4.7 ± 0.2	15.2 ± 0.2
C16:2	5.0 ± 0.1	6.8 ± 0.2	5.1 ± 0.1	ND
C16:3	4.0 ± 0.0	5.4 ± 0.1	5.3 ± 0.1	ND
C18:0	0.7 ± 0.0	7.8 ± 0.1	14.6 ± 0.4	5.5 ± 0.0
C18:1	5.8 ± 0.1	2.6 ± 0.0	*20.6* *±* *0.3*	10.3 ± 0.3
C18:2	2.1 ± 0.0	12.4 ± 0.1	10.5 ± 0.1	ND
C18:3	19.8 ± 0.2	12.2 ± 0.2	10.5 ± 0.1	ND
Others	8.5 ± 0.1	13.0 ± 0.2	20.1 ± 0.4	51.8 ± 1.2
Total saturated fatty acid	37.7	25.8	33.6	66.5
Monounsaturated fatty acid	31.0	31.4	26.8	33.5
Polyunsaturated fatty acid	31.3	42.8	39.6	ND
Total unsaturated fatty acid	62.3	74.2	66.4	33.5

Italic values indicate maximum relative abundance of fatty acids in the group

*ND* not detected

## Discussion

Mixotrophic cultivation is an attractive way of enhancing the concentration of algal biomasses (Yen and Chang 2013) and mode by which the microalgae can drive both photoautotrophy and heterotrophy and utilize both inorganic and organic carbon sources (Kang et al. 2004). In this study, a two-stage method of mixotrophic cultivation of *C. vulgaris* has been treated in detail. Phase I was used to improve the biomass concentration of the microalgae and Phase II to enhance the CO_2_ bio-fixation rate and lipid productivity.

By adding the natural antioxidant NaE and aerating the culture mixture with air in Phase I of the two-stage process, the oxidized intermediates from the NaE can be used by the *C. vulgaris* as organic carbon sources for mixotrophic growth (Cui et al. 2017). This is why the microalgal growth rates in the MX, MX-N, and MX-C samples were much greater than that in the PA group during the first 9 days (Fig. 2a), and why the average biomass productivity was 63.6% higher in those samples compared to the PA group (Table 3). As reported in the literature, by complementing the photoautotrophic process with organic substrates, the mixotrophic cultivation of microalgae can improve the growth rate, shorten the growth cycle, and increase biomass productivity (Park et al. 2012).

The biomass concentration of 1.66 g L^−1^ (day 9) achieved in this study is roughly 1.43 that of the biomass concentration of 1.16 g L^−1^ (day 12) reported in a previous study (Cui et al. 2017) (*p* < 0.05). In Cui et al.’s work, no aeration was used but the NaE dosage was the same (8 g L^−1^). This suggests that aeration is essential to improve the growth rate of *C. vulgaris* in a mixotrophic culture system (compared to photoheterotrophic cultivation). There are two reasons for this:

1. Aeration accelerates the oxidation of NaE. Subsequently, larger amounts of oxidative intermediate products are formed which can be utilized as organic carbon sources to facilitate mixotrophic growth of *C. vulgaris*. This is also illustrated by the TOC consumption data presented in Table 2. (In this work, TOC decreased by ~ 1630 mg L^−1^ in 9 days which should be compared to a decrement of 477 mg L^−1^ in 12 days in Cui et al. 2017 study.) The observed pH variation may also increase the credibility of this view. Acidic intermediate products are formed during NaE oxidation (Cui et al. 2017) which cause the pH in mixotrophic culture systems to decrease for the first 3 days. These products will be subsequently taken up by the *C. vulgaris* and used for oxidative assimilation via aerobic glycolysis. This results in an increase in pH from day 3 onwards and a decrease in the TOC level (Fig. 2b and Table 2).
2. Aeration provides O_2_ which contributes to the heterotrophic (aerobic respiration) part of the mixotrophic growth process. Yen and Zhang (2011) have suggested that low dissolved oxygen levels could retard cell growth, while high levels lead to rapid cell growth and maximum dry weights in heterotrophic cultures. Yeh and Chang (2012) also made a similar observation (*C. vulgaris* ESP-31 cultivated in a mixotrophic culture system produces a higher biomass than that produced using a photoheterotrophic culture system).

There was a rapid growth of *C. vulgaris* during the first 6 days (Fig. 2a). Table 2 shows that the consumption of NO_3_-N was ~ 94 mg L^−1^ in the three mixotrophic groups and ~ 51 mg L^−1^ in the photoautotrophic group. Both of these figures are larger than the initial NO_3_-N concentration in Bristol’s medium (47.49 mg L^−1^). Thus, it was necessary to replenish the supply of nitrogen on day 4 for all of the groups. Procházková et al. (2014) reported that the most widely used nitrogen source for microalgal cultivation is nitrate and so this source was adopted here.

Table 2 also suggests that there was enough TP available during the experiment, with the smallest amount of residual TP available at the end of the cultivation period occurring in the MX group of samples (equal to ~ 4.71 mg L^−1^). A general conclusion can be drawn from the molecular formula of the microalgal biomass: CO_0.48_H_1.83_N_0.11_P_0.01_ (Katiyar et al. 2017). This suggests that more nitrogen than phosphorus is needed for the growth of the microalgae. As a result, no phosphorus source was added during the whole of the cultivation process in this study.

A stationary phase was observed around day 8 in the three mixotrophic groups. Of course, a possible reason for this restricted microalgal growth is that the microalgae was suffering from a nitrogen deficiency. Hence, large amounts of nitrogen were added to the MX and MX-C samples on day 9 (that is, at the beginning of the second phase) and the MX-N samples were not changed so they could act as a nitrogen-deficient control. Meanwhile, air aeration of the MX and MX-N groups was changed to 10% CO_2_ aeration and the MX-C group was left unchanged to act as a CO_2_-deficient control.

As can be seen from Fig. 2b, aeration with a high concentration of CO_2_ (10%) lead to sharp decreases in the pH values of the MX and MX-N samples on day 10, and the acidic environment produced appears to have had a slightly inhibitory effect on the growth of the microalgae (Fig. 2a). Although the pH values of the MX-N samples tended to stabilize in subsequent days, nitrogen deficiency limited the growth of *C. vulgaris* (Table 2) in them. This lead to a 0.13 g L^−1^ decrement in the biomass in this group during the second cultivation stage compared to the end of Phase I (Table 3). Substantial growth, on the other hand, was observed in the MX samples (Fig. 2a) which may be attributed to the abundance of nutrient salts present.

Interestingly, both inorganic and organic carbon sources appear sufficient for the growth of *C. vulgaris*. The primary carbon source utilized for the growth of the algae was CO_2_ (TOC decreased by ~ 57 mg L^−1^ [Table 2) while the biomass increased by 1790 mg L^−1^ (Table 3)]. That is to say, photoautotrophic growth was the major form of *C. vulgaris* growth in the MX samples during Phase II. In contrast, the TOC fell by ~ 406 mg L^−1^ in the MX-C group during the second stage while the biomass of the microalgae increased by just 400 mg L^−1^. This indicates that the *C. vulgaris* in the MX-C group was still growing via mixotrophy, even though enough CO_2_ was not being supplied—that is, the carbon source used for growth at this time was still the organic carbon.

The two possible pathways for TOC utilization in Phase II of the MX-C group could be: (i) the previously oxidized acidic intermediates were further oxidized to CO_2_ which was then released into the culture system and subsequently trapped/reused for photosynthesis; (ii) the intermediates are utilized directly by the *C. vulgaris* for heterotrophic growth; the CO_2_ released by the microalgae via aerobic respiration could be then utilized in the same way as in pathway (i) (Mata et al. 2010; Li et al. 2014). This could possibly explain why the TOC decrement exceeds the increment of carbon content in the biomass produced in MX-C. However, the relative importance of the two pathways needs to be studied further. On the whole, CO_2_-deficiency is an important limitation on the further growth of *C. vulgaris* in the MX-C group of samples even though an abundant source of nitrogen was available.

One of the aims of our study was to take advantage of the mixotrophic growth of *C. vulgaris* in the first stage of the procedure to obtain a high biomass concentration in a short time and then permit the microalgae to bio-fix much more of the CO_2_ available in the second stage. This goal was clearly achieved as the increase in biomass concentration in MX in Phase II within 8 days was higher than the biomass increment in the PA group during the whole of its period of cultivation (1790 vs. 1210 mg L^−1^, see Table 3). However, it is difficult, in general, to calculate the rate of CO_2_ bio-fixation during mixotrophic cultivation. The determination result shows that the carbon content of the *C. vulgaris* is 50.4%. Assuming that all of the TOC decrement is utilized for *C. vulgaris* growth, then, for Phase II of the MX samples, the rate of bio-fixation of CO_2_ by *C. vulgaris* must be at least 344 mg CO_2_ L^−1^ day^−1^. This is roughly 1.8 times that occurring in PA during the whole of its cultivation period (124 mg CO_2_ L^−1^ day^−1^). Consequently, the two-stage mixotrophic cultivation process is an effective way of obtaining a high biomass concentration of *C. vulgaris* and improving its CO_2_ bio-fixation rate.

We further note that microalgal lipid accumulation and production during these kinds of two-stage cultivation procedures has rarely been investigated. Cheirsilp and Torpee (2012) found that the lipid content of four microalgal strains did not differ significantly using three different cultivation modes (although the growth of all strains was improved under mixotrophic conditions). This conclusion can also be drawn from the present study as the lipid content of the 4 groups ranged from 20.19 to 24.63%. The maximum lipid content was observed in the photoautotrophic group, in agreement with the results reported by Liang et al. (2009) and Heredia-Arroyo et al. (2011) who also focused on *C. vulgaris*. Interestingly, N limitation in MX-N groups hasn’t improved the lipid synthesis dramatically compared to N replete groups (MX) (Table 3) as many previous studies reported (Deng et al. 2011; Mujtaba et al. 2012). However, many microalgae species have been found to obtain higher lipid contents at N replete instead of N deplete conditions. Feng et al. (2011) reported the same results, *C. pyrenoidosa* had the maximum lipid content and productivity under the N replete medium rather than N deplete medium. And the reason was owing to the enhanced activity of acetyl-CoA carboxylase or other key enzymes related to the conversion of poly saccharides into lipids (Feng et al. 2011; Bellou et al. 2014). According to the investigation by Griffiths and Harrison (2009), 17 of 24 different microalgal species showed lipid accumulation under N deplete conditions while the others showed an increase of lipid content under N deplete conditions. So it is thought that the responses of algal cells to nitrogen deplete/replete conditions are species-specific as well as an intrinsic characteristic (Kim et al. 2016). And maybe the same reason could be used to explain this interesting result in the present study. Futhermore, the two-stage, nutrient-supplemented, mixotrophic cultivation process (group MX) showed the highest levels of lipid production and productivity (786.60 mg L^−1^ and 43.70 mg L^−1^ day^−1^, see Table 3). This implies that two-stage mixotrophic cultivation is also a highly efficient way of improving the quantity of lipids produced.

The fuel properties of biodiesel are strongly influenced by the properties of the individual fatty esters in biodiesel. PUFAs were observed in all three of the mixotrophic groups (Table 4). This is presumably because the added NaE, a deoxidant, prevented the PUFAs from being oxidized to MUFAs. The increase in PUFA concentration can reduce the viscosity, leading to better lubricity compared to saturated fatty acids (Serrano et al. 2014). This can enhance cold flow performance due to the fact that the presence of unsaturated FAs retards the crystallization process at low temperature (Kim et al. 2016). Although relatively high levels of PUFAs would affect the oxidative stability, since the oxidation rates of C16:2, C16:3 and C18:2, C18:3 are higher compared to those of C16:1 and C18:1. The problems can be solved by adding antioxidants, such as NaE present in this study. That C16 and C18 account for the majority of the carbon chain lengths featuring in the FAs is in accord with other results reported in the literature and suggests that the main FAs present in the lipids derived from *Chlorella* sp. are essentially short-chain FAs (C14–C18) (Huang et al. 2010). In the present study, over 60–68% of the lipids produced consist of TSFAs and MUFAs, which are considered to be suitable for synthesizing biodiesel (Sheehan et al. 1998). Hence, the two-stage mixotrophic cultivation process also seems to be a highly suitable way of generating biodiesel feedstock using *C. vulgaris*.

Moreover, linolenic and linoleic acids are known to be essential FAs and are an obligatory dietary requirement for humans and animals alike (Sinclair 1990). Most humans have diets that are deficient in linolenic acids and PUFAs are in great demand as dietary supplements and also for aquaculture. Therefore, microalgal oils derived via two-stage mixotrophic cultivation may be an excellent source of such high-value products.
